# Targeting α- and β-Adrenergic Receptors Differentially Shifts Th1, Th2, and Inflammatory Cytokine Profiles in Immune Organs to Attenuate Adjuvant Arthritis

**DOI:** 10.3389/fimmu.2014.00346

**Published:** 2014-08-11

**Authors:** Cheri L. Lubahn, Dianne Lorton, Jill A. Schaller, Sarah J. Sweeney, Denise L. Bellinger

**Affiliations:** ^1^College of Arts and Sciences, Kent State University, Kent, OH, USA; ^2^Department of Pathology and Human Anatomy, Loma Linda University School of Medicine, Loma Linda, CA, USA

**Keywords:** sympathetic nervous system, rheumatoid arthritis, macrophage, T cell, cytokine balance, spleen, lymph nodes, peripheral blood mononuclear cells

## Abstract

The sympathetic nervous system (SNS) regulates host defense responses and restores homeostasis. SNS-immune regulation is altered in rheumatoid arthritis (RA) and rodent models of RA, characterized by nerve remodeling in immune organs and defective adrenergic receptor (AR) signaling to immune cell targets. The SNS typically promotes or suppresses inflammation via α- and β_2_-AR activation, respectively, and indirectly drives humoral immunity by blocking Th1 cytokine secretion. Here, we investigate how β_2_-AR stimulation and/or α-AR blockade at disease onset affects disease pathology and cytokine profiles in relevant immune organs from male Lewis rats with adjuvant-induced arthritis (AA). Rats challenged to induce AA were treated with terbutaline (TERB), a β_2_-AR agonist (600 μg/kg/day) and/or phentolamine (PHEN), an α-AR antagonist (5.0 mg/kg/day) or vehicle from disease onset through severe disease. We report that in spleen, mesenteric (MLN) and draining lymph node (DLN) cells, TERB reduces proliferation, an effect independent of IL-2. TERB also fails to shift T helper (Th) cytokines from a Th1 to Th2 profile in spleen and MLN (no effect on IFN-γ) and DLN (greater IFN-γ) cells. In splenocytes, TERB, PHEN, and co-treatment (PT) promotes an anti-inflammatory profile (greater IL-10) and lowers TNF-α (PT only). In DLN cells, drug treatments do not affect inflammatory profiles, except PT, which raised IL-10. In MLN cells, TERB or PHEN lowers MLN cell secretion of TNF-α or IL-10, respectively. Collectively, our findings indicate disrupted β_2_-AR, but not α-AR signaling in AA. Aberrant β_2_-AR signaling consequently derails the sympathetic regulation of lymphocyte expansion, Th cell differentiation, and inflammation in the spleen, DLNs and MLs that is required for immune system homeostasis. Importantly, this study provides potential mechanisms through which reestablished balance between α- and β_2_-AR function in the immune system ameliorates inflammation and joint destruction in AA.

## Introduction

Autonomic and immune dysfunction, and dysregulation of their cross-communication in autoimmune diseases, are hallmarks of many autoimmune diseases, including rheumatoid arthritis (RA) (1–4). In RA, chronic inflammation, “self” targeted arthritogenic T cells and autoantibodies drive the damage in the affected joints and visceral organs (5). Thus, altered innate and adaptive immunity are key mediators of the disease process (5, 6). Dysregulation of the immune system is coupled with hyperactivity of the sympathetic nervous system (SNS). Dysregulation of the SNS in the immune system contributes significantly to disease onset, and greater severity in RA and experimentally-induced arthritis (7, 8). The contribution of a dysregulated SNS and the altered cross-communication between SNS and the immune system in autoimmune diseases is complex. In adjuvant- and collagen-induced arthritis models [adjuvant-induced arthritis (AA) and CIA, respectively], the SNS exerts a pro-inflammatory influence during the inductive, asymptomatic phase, but suppresses the inflammatory and cell-mediated response after disease onset (9, 10). Thus, we still do not understand the complex and diverse roles of the SNS in regulating innate and adaptive immunity that mediate rheumatic disease mechanisms prior to and after disease onset. Growing evidence indicates that the SNS is an important target for developing disease amelioration, or even preventing disease onset, supporting the need to understand sympathetic-immune system cross-talk in RA (2).

We have shown disease promoting effects of sympathetic innervation of secondary lymphoid organs during the induction phase, a time of antigen processing, and disease enhancing effects in the effector phase of AA (11, 12). This finding was subsequently confirmed using the collagen-induced arthritis model of RA (13). In these studies, sympathetic nerves were destroyed by selective toxins (11, 13) or β_2_-adrenergic receptor (AR) agonist and/or α-AR antagonist treatment (12) prior to the autoimmune inducing challenge or after disease onset. Treatment with a β_2_-AR agonist and/or α-AR antagonist shifts the balance of pro- and anti-inflammatory cytokine production toward increased disease severity if administered prior to or reduced disease activity after disease onset, respectively. These findings indicate that changes in the nerves and receptors contribute to the opposite disease outcome effects of the SNS observed after challenge and disease onset. Consistent with this notion, we (14–16) and others (17) previously reported dramatic changes in the density and distribution of sympathetic nerves to discrete compartments in secondary immune organs in rodent models of RA, and in the affected joints of RA patients. These anatomical changes suggest that sympathetic nerves interact with various immune cell populations in different activational states and functional compartments. They also implicate a link between consequent changes in sympathetic-immune cell signaling with disease development and progression.

The SNS regulates the functions of immune cells that mediate rheumatic disease through the activation of α- and β_2_-ARs expressed on their cell surfaces (10, 17–19). Sympathetic nerves target immune cells in secondary lymphoid organs (15, 16), and the immune cell infiltrates and tertiary lymph nodules that occur in the affected joints (20). In secondary lymphoid organs, neurotransmitters, predominantly norepinephrine released from sympathetic nerves, activate β_2_-ARs expressed in T cells to inhibit IL-2 production, which subsequently suppresses lymphocyte proliferation required for clonal expansion [reviewed in Ref. (21)]. Activation of β_2_-ARs also inhibit cellular and promote humoral immunity by regulating the phenotypic differentiation of CD4+ T helper (Th) cells in response to challenge with T-cell-dependent antigens (21). This occurs by norepinephrine activating Th0/Th1 cell β_2_-ARs, which increase intracellular cAMP production. Cyclic AMP then inhibits IFN-γ production. The decrease in IFN-γ reduces the inhibitory cross-regulation of IFN-γ on IL-4 production, thus promoting IL-4 synthesis by Th2 cells. In this manner, the SNS provides a negative feedback mechanism to restore immune system homeostasis after antigen challenges that activate cellular immunity (21).

Recently, we found that activation of β_2_-ARs no longer induces cAMP in immune cells in the spleen and lymph nodes that drain the arthritic hind-limbs in AA during severe disease (18). Altered β_2_-AR responses occurred concomitantly with altered receptor-ligand affinity and lymphoid tissue changes in receptor phosphorylation. These findings predict that a negative feedback mechanism required to restore immune system homeostasis after adjuvant challenge is lost after disease onset. However, the impact of sympathetic regulation of IL-2, IFN-γ, and IL-4 production in disease-relevant secondary lymphoid organs has not been determined.

The SNS also time-dependently regulates pro- and anti-inflammatory cytokine production relative to the antigen challenge [reviewed in Ref. (21)]. After antigen challenge, norepinephrine released from sympathetic nerves at the site of challenge promotes TNF-α and suppresses IL-10 production by activating α-ARs expressed in macrophages to amplify the ensuing inflammatory response. Similarly, norepinephrine promotes TNF-α and suppresses IL-10 production in dendritic cells from secondary lymphoid tissue via α-ARs. In contrast, activation of β_2_-AR in macrophages and dendritic cells suppresses TNF-α and promotes IL-10 production. The balance between the anti- and pro-inflammatory effects of the SNS is dependent upon the balance between α- and β_2_-AR expression, intracellular signaling pathways they activate and the dynamic local environment that follows the arthritis inducing challenge (9, 12). In this manner, the SNS regulates the initiating and shutting off of inflammatory and innate immune responses. In animal models of RA, α-AR antagonists and β_2_-AR agonists promote or inhibit joint inflammation if administered prior to or after disease onset, respectively. The effects of altered sympathetic innervation and changes in β_2_-AR function on SNS regulation of pro- and anti-inflammatory cytokines seen previously in secondary lymphoid organs of arthritic rats have not been examined.

The purpose of this study was to determine how the SNS affects *ex vivo* cytokine production in secondary lymphoid organs during the effector phase of disease. Specifically, we examined the effect of AR selective drug treatments in (1) modulating IL-2 and proliferation, and (2) the balance between IFN-γ and IL-4 and between TNF-α and IL-10 after AA development. A specific β_2_-AR agonist and an α-AR antagonist, alone and in combination [previously designated SH1293 (12)] were administered *in vivo* after disease onset. These treatments were used to determine the contribution of each receptor subtype in altering cytokine production and disease outcome. T cell and macrophage cytokines were measured *ex vivo* for each tissue collected from each treatment group. Cytokines with crucial roles in Th cell differentiation and clonal expansion or inflammation were selected for assessment: (1) immune cell production of IL-2, an important cytokine for development and differentiation of Th cells and proliferative responses required for clonal expansion (22); (2) IL-4 and IL-10, cytokines, which promote Th2 cell development and have anti-inflammatory functions; (3) IFN-γ and TNF-α, which promote Th1 cell development and which drive inflammation (23, 24).

## Materials and Methods

### Supplies, drugs, and adjuvant preparation

All tissue culture media and supplements were obtained from Gibco BRL (Rockville, MD, USA) unless otherwise stated. OPTIA sandwich ELISA kits for IL-2, IL-4, IL-10, IFN-γ, and TNF-α were purchased from BD Pharmingen (San Diego, CA, USA). The non-specific α-AR antagonist, phentolamine, and the β_2_-AR agonist, terbutaline, were obtained from Sigma Chemical Company (St. Louis, MO, USA). All adrenergic drugs were dissolved in 0.01 mM ascorbic acid in 0.9% sterile, endotoxin-free saline. Complete Freund’s adjuvant (CFA; 0.03 g dried and heat-killed *Mycobacterium butyricum*; Difco, Detroit, MI, USA) was emulsified in 10 ml sterile mineral oil, as previously described (18). The suspension was treated with a sonic dismembraner for 5 min to keep the bacterial cell wall in suspension for animal injections. A single preparation of CFA was used to minimize variability, and 100% of the animals developed arthritis.

### Animals

Male Lewis rats (200–250 g) were obtained from Charles River Laboratories (Raleigh, NC, USA) and housed two per cage for 3 weeks prior to the start of each experiment. The animals were maintained on a 12-h off/on light schedule, and food (Purina Lab Diet 5001) and water were available *ad libitum*. For AA rats, the food was placed in the bottom of the cage, and water was supplied using long-stemmed sipper tubes. All rats were observed to eat and drink throughout the study period. Animals were weighed biweekly to monitor adequate weight gain. All animals were treated in the same manner. Other than the development of arthritis, rats were healthy throughout the experiment. All protocols for the use and care of the animals in the study were approved by our Animal Use and Care Committee, and complied with NIH guidelines for the humane use and care of research animals.

Thirty-two male Lewis rats were given 100 μl of CFA by intradermal injection into the base of the tail. Twelve days later, rats were randomly assigned to one of four groups (*N* = 8 per group): (1) phentolamine [PHEN; 125 μg/day intraperitoneal (i.p.)]; (2) terbutaline [TERB; 1200 μg/day (i.p.)]; (3) phentolamine and terbutaline (PT, same dose as PHEN and TERB); or (4) vehicle (VEH). Drug treatment was divided into two injections in a total volume of 250 μl per injection given at 7 a.m. and 6 p.m., as previously described (12). This treatment regimen was based on previous reports demonstrating predictable pharmacological effects on disease severity in arthritic rats (9, 12). Adrenergic therapies began 12 days after immunization, the time of disease onset, and continued until sacrifice. All animals were weighed prior to sacrifice on day 28 using an overdose of 8% chloral hydrate 10.0 ml/kg body weight.

At sacrifice, the inguinal and popliteal lymph nodes (DLN, lymph nodes that drain the site of antigen challenge), mesenteric lymph nodes (MLN), and spleen were dissected and weighed, and peripheral blood was collected. Cell suspensions from all immune tissues were prepared for cell culture. All methods used in this study have been previously described in detail (12, 18). Non-arthritic animals were not included in this study, but reference ranges from non-arthritic rats from our database are shown as light gray horizontal bars to indicate normal values or non-specific activity, as appropriate.

### Assessment of disease outcome

The inflammatory response in the arthritic rats was assessed by routine methods previously described (12, 18). Dorsoplantar widths of the hind feet were measured using a Mitutoyo Corporation dial thickness gage on the day of sacrifice. Prior to sacrifice, radiographs of the hind-limbs were taken to assess disease severity. Radiographs were taken using the following settings: 400 nN, 50 kVp, 0.4 s exposure time at 40 cm, using an X-OMAT processor. Using a grading scale modified from Ackerman and coworkers (25), the radiographs were coded to obscure the treatment groups, then two independent observers subjectively rated each of the radiographs on the scale: 0 (normal), 1 (slight), 2 (mild), 3 (moderate), and 4 (severe) abnormalities in the tissue. The radiographs were scored for each of the following characteristics: (1) soft tissue swelling as indicated by the width of soft tissue shadows and alterations in the normal configuration of the soft tissue planes; (2) osteoporosis (recognized by increases in radiolucency relative to uninvolved adjacent bone); (3) cartilage loss shown by narrowing of the joint spaces; (4) heterotopic ossification defined as proliferation of new bone tissue (fine ossified line paralleling normal bone, but not contiguous with calcified area of the bone itself); and (5) bone erosions. The radiographic scores for each category were added for both hind-limbs giving a maximum score of 40.

### Immune cell cultures

Spleens were placed in a stomacher bag and homogenized in Hank’s balanced salt solution (HBSS) for 30 s. Spleen cells were washed with HBSS, and passed through a nylon mesh (Fisher Scientific, St Louis, MO, USA) to remove the connective tissue. Spleen cells were centrifuged and the pellet was resuspended in 5 ml NH_4_Cl hypotonic buffer for 3 min to lyse red blood cells. The cells were washed 2× with 10 ml HBSS, centrifuged, and resuspended in complete RPMI 1640 media (Gibco BRL, Rockville, MD, USA) supplemented with 5% fetal calf serum and 1% antibiotic/antimycotic (complete media). Using forceps, MLNs and DLNs were teased apart in HBSS. Each homogenate was passed through a nylon mesh, washed in 5 ml HBSS, centrifuged, and resuspended into complete media, as described above. Lymph node and spleen cells were counted using a hemocytometer and adjusted to 2 × 10^6^ cells/ml in complete media.

Blood was collected using cardiac puncture into 7-ml lithium heparin vacutainer tubes. After tube inversion (7×), blood cells were pelleted at 1,000 rpm for 15 min at 10°C. The buffy coat was removed and placed into 10 ml NH_4_Cl hypotonic buffer for 3 min to lyse red blood cells. Peripheral blood mononuclear cells (PBMCs) were centrifuged, and the cell pellet was resuspended and washed 3× in 10 ml HBSS. Following the last centrifugation, the cells were resuspended in complete media. PBMCs were counted and suspended at 2 × 10^6^ cells/ml in complete media. In this study, additional immune challenge was omitted to better mimic *in vivo* disease conditions.

### *Ex vivo* cellular proliferation

Immune cells in supplemented complete RPMI 1640 were plated in 96-well, flat-bottom plates (Falcon, Oxnard, CA, USA) at 2 × 10^5^ cells/well in triplicate without further stimulation. In this study, additional immune challenge was omitted to better mimic *in vivo* disease conditions. Cultures were maintained for 72 h in a humidified, 7% CO_2_ incubator at 37°C. [^3^H]Thymidine (0.5 μCi/10 μl; Amersham, Arlington Heights, IL, USA) was added the last 24 h of culture. Cells were harvested onto glass fiber filters (Brandel, Gaithersburg, MD, USA) using a cell harvester (Brandel, Gaithersburg, MD, USA). The filters were placed in 5 ml of scintillation fluid (Fisher Scientific, Tustin, CA, USA), and [^3^H]Thymidine incorporation was determined using a liquid scintillation counter (Beckman, Brea, CA, USA).

### Cytokine production

Two milliliters of each cell suspension were plated into 24-well plates (Falcon, Oxnard, CA, USA), and incubated in 7% CO_2_ at 37°C for 24 h. After 24 h, the supernatants were harvested and stored at −80°C. Cytokine ELISAs were run according to the manufacturer’s instructions. Cytokine levels in unknown samples were determined through comparison with a standard curve obtained from known concentrations of the cytokines run in duplicate on each plate using a Ceres 900 HDI plate reader at 450 nm (Bio Tek Instruments Incorporated, Winooski, VT, USA).

### Statistical analysis

All data are expressed as a mean ± standard error of the mean (SEM), unless otherwise stated. For disease outcome measurements the right and left footpads from each animal were averaged, and the individual means per group determined (mean of a mean in millimeters). Group differences were determined by two-way ANOVA with repeated measures. Means found to be significantly different (*p* < 0.05) were subjected to Bonferroni *post hoc* analyses. Mean radiograph scores from two scorers completed without knowledge of the treatment group were averaged for each group, and then subjected to Kruskal–Wallis statistical analysis with Dunn *post hoc* testing. Body and spleen weights were averaged for each treatment group (mean expressed in grams).

For cell proliferation, counts from triplicate wells were averaged after subtraction of background radioactivity. Group means were determined, and data were expressed as [^3^H]thymidine incorporation in counts per minute (cpm). Cytokine concentrations from duplicate wells were averaged, group means were calculated, and the data expressed as a mean in picograms per milliliter. The individual cytokine and the cytokine ratios were determined for each animal by comparing the samples to the standard curve on each plate. Group differences in body and spleen weights, cytokine production, cytokine ratios, and cellular proliferation were determined by one-way ANOVA. Means found to be significantly different (*p* < 0.05) were subjected to Bonferroni *post hoc* analyses.

## Results

### Adrenergic drugs attenuated inflammation and bone loss

**Figure 1:**
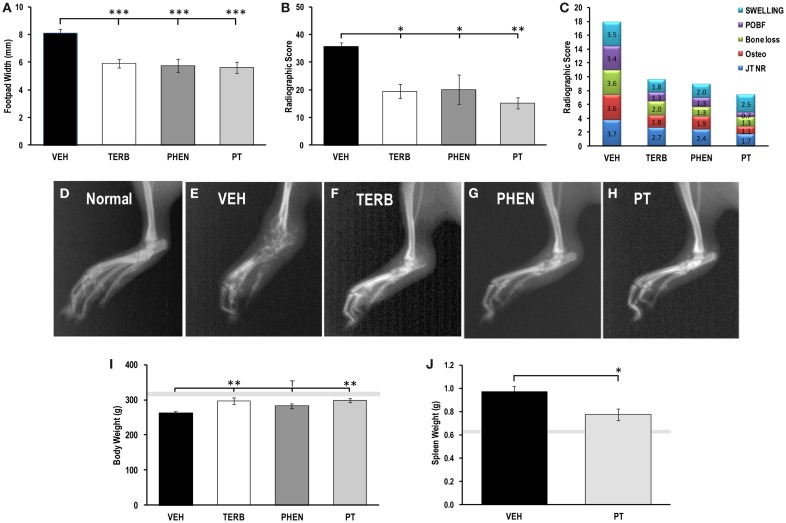
**Indices of disease severity**. Animals were treated with twice-daily i.p. injections of vehicle (VEH, black bars), terbutaline (TERB), phentolamine (PHEN), or phentolamine and terbutaline (PT) initiated 12 days after adjuvant challenge. **(A)** Mean Footpad widths ± SEM were significantly reduced in TERB (white bar), PHEN (dark gray bar), and PT (light gray bar) treated compared with VEH (black bar) treated rats. Light gray horizontal bar represents the mean footpad width ± SEM of naïve rat. *N* = 8. ***p < 0.001. **(B)** Mean radiographic scores ± SEM for, AA rats treated with TERB (white bar), PHEN (dark gray bar), and PT (light gray bar) were decreased compared with VEH (black bar). *N* = 8. *p < 0.05; **p < 0.01. **(C)** Stack-plots showing the contribution of each radiographic score component to the mean total radiographic score for rats treated with VEH, TERB, PHEN, or PT. Swelling, blue; joint narrowing (JT NR), red; osteoporosis, green; bone loss, purple; periosteal bone formation (POBF), turquoise. **(D)** Radiograph of the hind limb representative of normal untreated rats. **(E)** Radiograph of the hind limb representative of vehicle (VEH)-treated rats. **(F)** Radiograph of the hind limb representative TERB-treated rats. **(G)** Radiograph of the hind limb representative of PHEN-treated rats. **(H)** Radiograph of the hind limb representative of PT-treated rats. **(I)** Mean body weights in grams (g) ±SEM for rats treated with vehicle (VEH), terbutaline (TERB), phentolamine (PHEN), or phentolamine and terbutaline (PT). The light gray bar represents the range of body weights for untreated non-arthritic rats. *N* = 8. **p < 0.01. **(J)** Mean spleen weights in grams (g) ±SEM for rats challenged with adjuvant and treated with vehicle (VEH) or phentolamine and terbutaline (PT). The light gray bar represents the range for normal spleen weights of untreated non-arthritic rats. *N* = 8. *p < 0.05.

Footpad widths and X-ray scores from drug- and VEH-treated rats are presented in Figures 1A,B, respectively. All adrenergic interventions significantly reduced the dorsoplantar footpad width compared with VEH-treated arthritic rats (*p* < 0.001) (Figure 1A). All arthritic rats had greater footpad widths compared with non-AA rats (normal range indicated by the gray horizontal bar shown in Figure 1A). There was also a dramatic reduction in the radiographic scores from the arthritic rats treated with TERB compared with VEH-treated arthritic rats (Figure 1B; *p*_TERB_ < 0.05, *p*_PHEN_ < 0.05, or *p*_PT_ < 0.01). X-rays of ankle joints from VEH-treated arthritic rats showed greater soft tissue swelling and joint destruction compared with arthritic rats treated with TERB, PHEN, or PT (Figures 1D–H). These observations were confirmed with X-ray analysis (Figure 1C). Figure 1C demonstrates the adrenergic drug-induced reduction in the components that comprise the radiographic scores. Adrenergic drug treatments reduced periosteal bone formation (POBF) 260–490%, bone erosions 170–270%, osteoporosis 190–320%, cartilage loss (JT NR) 140–220%, and soft tissue swelling 140–290% compared with VEH-treated arthritic animals. The rank order of potency for reducing soft tissue swelling and lower X-ray scores for the treatments was PT > TERB = PHEN > VEH.

### Adrenergic drugs prevented loss of body weight and increased spleen weight

Between day 8 and 12 post-CFA challenge, there was a sharp decline in body weight (~10–15%) that leveled off between days 12 and 16, and then was maintained through the effector phase of the disease (data not shown). In rats treated with TERB and PT, weight loss was prevented compared with VEH-treated control animals at day 28 post-CFA challenge (Figure 1I, *p* < 0.01). There was a trend (*p* < 0.1) toward weight loss prevention in rats treated with PHEN. Additionally, disease development resulted in greater spleen weights compared with the normal range (light gray horizontal bar; *p* < 0.001; Figure 1J). Combined drug treatment, PT, partially reversed the disease-induced increase in spleen mass compared with the VEH-treated AA rats (*p* < 0.05); however, the PT-treated animals still had spleens with significantly greater mass than the non-arthritic controls (light gray horizontal bar; *p* < 0.05).

### No effects of adrenergic drugs on PBMC proliferation despite lower IL-2 production by α-AR blockade

**Figure 2:**
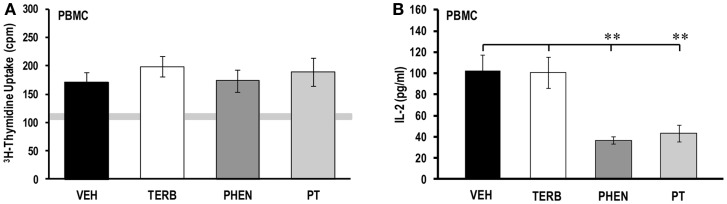
**Proliferative response and IL-2 production in peripheral blood mononuclear cells (PBMC)**. Animals were treated with twice-daily i.p. injections of vehicle (VEH, black bars), terbutaline (TERB, white bars), phentolamine (PHEN, dark gray bars), or phentolamine and terbutaline (PT, light gray bars) initiated 12 days after adjuvant challenge. **(A)** Proliferative responses 72 h post-PBMC cell culture did not differ between treatment groups. *N* = 8. Gray horizontal bar represents the range for ^3^H-thymidine incorporation in unstimulated PBMCs. **(B)** IL-2 production 24 h-post PBMC culture were reduced in PHEN- and PT-treated compared with VEH-treated AA rats. *N* = 8. All data represent means ± SEM. **p < 0.01.

On day 28 post-CFA challenge, the proliferative activity of PBMCs from arthritic rats (Figure 2A) was low in all the treatment groups (~130–200 cpm), but was slightly higher than the level seen in untreated PBMCs from non-AA rats (light gray horizontal bar). There were no differences in PBMC proliferation among the drug treatment groups or when compared with VEH treatment. Despite the absence of drug-induced effects on PBMC proliferative responses, *ex vivo* IL-2 production was affected by drug treatment on day 28 post-CFA challenge (Figure 2B). PBMCs from VEH- and TERB-treated arthritic rats released relatively similar levels of IL-2 (Figure 2B). Unlike TERB, IL-2 concentration was reduced in both PHEN- (*p* < 0.01) and PT- (*p* < 0.01) treated PBMC cultures compared with VEH-treated AA controls.

### α-AR adrenergic therapies reduced PBMC Th1 and pro-inflammatory cytokine production

**Figure 3:**
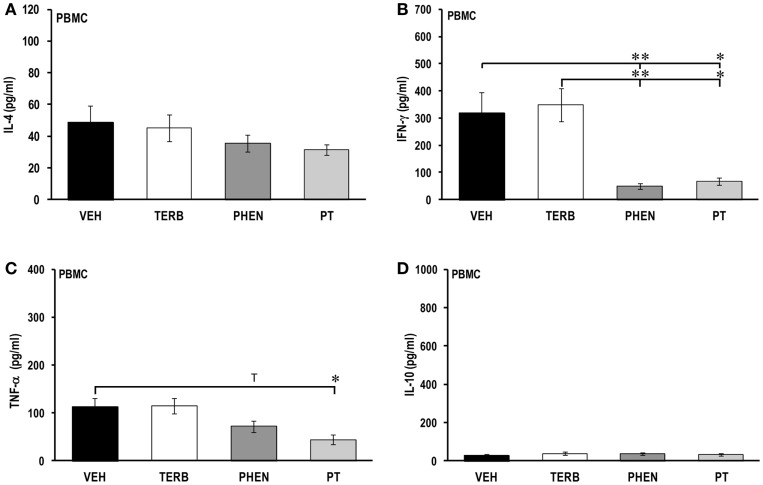
**Cytokine production in peripheral blood mononuclear cells (PBMC)**. Animals were treated with twice-daily i.p. injections of vehicle (VEH, black bars), terbutaline (TERB, white bars), phentolamine (PHEN, dark gray bars), or phentolamine and terbutaline (PT, light gray bars) initiated 12 days after adjuvant challenge. **(A)** IL-4 concentrations 24 h after PBMC culture did not differ among the treatment groups. *N* = 8. **(B)** IFN-γ production 24 h post-PBMC culture was reduced by either PHEN or PT compared with VEH treatment. *N* = 8. *p < 0.05; **p < 0.01. **(C)** TNF-α secretion from PBMCs was reduced by PT compared with VEH treatment 24 h post-culture. *N* = 8. *p < 0.05. **(D)** IL-10 production in PBMCs was similar in all treatment groups after 24 h of cell culture. *N* = 8.

In *ex vivo* cultures of PBMCs from all treatment groups, IL-4 concentrations were similar (Figure 3A). PBMCs from VEH-treated arthritic rats secreted more IFN-γ (319 ± 76 pg/ml) (Figure 3B) compared with IL-2 (103 ± 15 pg/ml, Figure 2B) and IL-4 (49 ± 11 pg/ml, Figure 3A). Treatment with TERB alone did not affect IFN-γ secretion. However, PHEN and PT significantly reduced the IFN-γ concentration compared with VEH- or TERB-treated AA rats (PHEN vs. VEH or TERB, *p* < 0.01; PT vs. VEH or TERB, *p* < 0.05).

PBMCs from VEH-treated arthritic rats secreted low levels of TNF-α (123 ± 21 pg/ml) (Figure 3C) and IL-10 (28 ± 5 pg/ml) (Figure 3D). There was no difference in TNF-α production in TERB- or VEH-treated arthritic rats (Figure 3C). However, PHEN treatment showed a trend (*p* < 0.1) toward reduced TNF-α production compared with VEH-treated arthritic rats (Figure 3C) and PT treatment significantly inhibited TNF-α release compared with VEH- or TERB-treated arthritic rats (*p* < 0.05) (Figure 3C). Treatment with TERB, PHEN, or PT had no effects on IL-10 concentration compared with VEH-treated arthritic rats (Figure 3D).

### Adrenergic therapies reduced *ex vivo* spleen cell proliferative response and differentially affected IL-2 production

**Figure 4:**
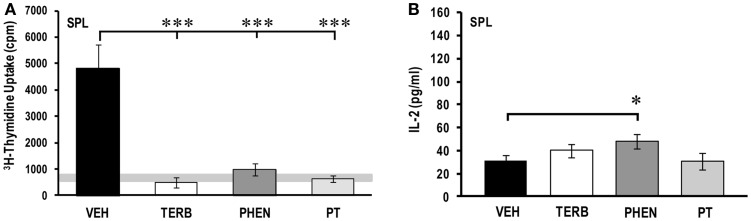
***Ex vivo* proliferation and IL-2 production by spleen (SPL) cells 72 and 24 h post-culture, respectively**. Animals were treated with twice-daily i.p. injections of vehicle (VEH, black bars), terbutaline (TERB, white bars), phentolamine (PHEN, dark gray bars), or phentolamine and terbutaline (PT, light gray bars) initiated 12 days after adjuvant challenge. **(A)** ^3^H-Thymidine incorporation into spleen cells was significantly lower in all drug-treated groups compared with VEH controls. The horizontal light gray bar represents the range of non-specific background in untreated non-arthritic rats. *N* = 8. ***p < 0.001. **(B)** IL-2 secreted by spleen cells was greater with PHEN compared with VEH treatment, but IL-2 concentrations in the other treatments did not differ from VEH-treated rats. *N* = 8. *p < 0.05.

Thymidine incorporation was greater in the spleen cells from VEH-treated arthritic than untreated non-arthritic rats (light gray horizontal bar; Figure 4A). Splenocyte proliferation was 28-fold greater than PBMC proliferation (Figure 2A). *In vivo* treatment with TERB, PHEN, or PT markedly reduced spleen cell proliferation compared with VEH treatment (Figure 4A; *p* < 0.001). IL-2 levels were low (28 ± 5 pg/ml), but detectable in the VEH-treated arthritic animals (Figure 4B). Despite the drug-induced suppression in proliferation (Figure 4A), IL-2 concentrations (Figure 4B) were no different in cultures from TERB- or PT-treated rats, and elevated in cultures from PHEN-treated rats compared with VEH treatment (*p* < 0.05).

### Adrenergic therapy reduced *ex vivo* pro-inflammatory and increased Th2 cytokine production in spleen cells

**Figure 5:**
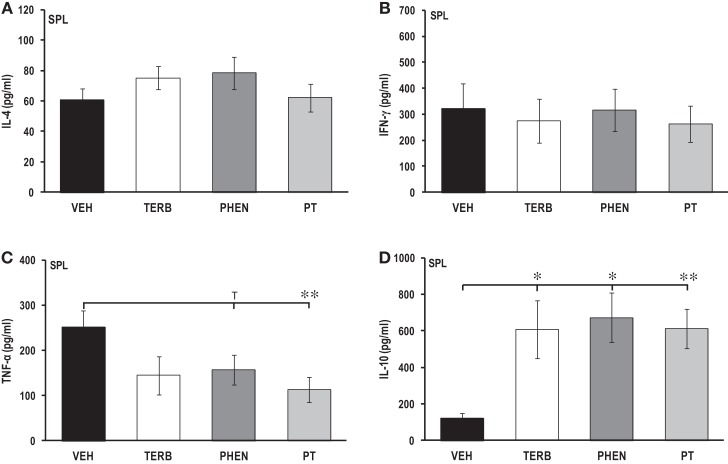
**Cytokine production by spleen (SPL) cells after 24 h of culture**. Animals were treated with twice-daily i.p. injections of vehicle (VEH, black bars), terbutaline (TERB, white bars), phentolamine (PHEN, dark gray bars), or phentolamine and terbutaline (PT, light gray bars) initiated 12 days after adjuvant challenge. **(A)** IL-4 production. *N* = 8. **(B)** IFN-γ levels. *N* = 8. **(C)** TNF-α secretion. *N* = 8. **p < 0.01. **(D**) IL-10 release. *N* = 8. *p < 0.05; **p < 0.001.

Figures 5A–D show the *ex vivo* secretion of IL-4 (Figure 5A), IFN-γ (Figure 5B), TNF-α (Figure 5C), and IL-10 (Figure 5D). There was no effect of any of the drug treatments on IL-4 or IFN-γ production compared with VEH controls (Figures 5A–B). Treatment with PT significantly reduced TNF-α production by spleen cells compared with VEH-treated arthritic rats (Figure 5C; *p* < 0.01). Spleen cells from rats receiving PHEN tended to produce less TNF-α than rats treated with VEH (*p* < 0.1), while TERB had no effect compared to VEH treatment. Spleen cells from VEH-treated arthritic rats secreted ~4-fold more IL-10 (Figure 5D) compared with PBMCs (Figure 3D). Interestingly, TERB, PHEN, or PT treatment markedly enhanced splenocyte IL-10 production compared with VEH-treated arthritic rats (Figure 5D; *p*_TERB_ < 0.05; *p*_PHEN_ < 0.05; *p*_PT_ < 0.01). There were no significant differences in splenocyte IL-10 production between any of the adrenergic drug-treated animals.

### Adrenergic therapies suppressed *ex vivo* cell proliferation in DLN, but differentially affected IL-2 production

**Figure 6:**
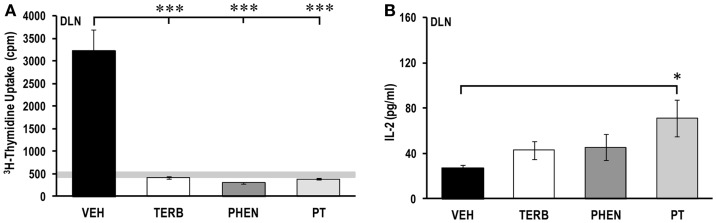
**Proliferation of draining lymph node (DLN) cells and IL-2 production**. Animals were treated with twice-daily i.p. injections of vehicle (VEH, black bars), terbutaline (TERB, white bars), phentolamine (PHEN, dark gray bars), or phentolamine and terbutaline (PT, light gray bars) initiated 12 days after adjuvant challenge. **(A)** All drug treatments suppressed proliferation of DLN cells compared with VEH treatment. Horizontal light gray bar demonstrates non-specific background. **(B)** IL-2 production by DLN cells is elevated in PT compared with VEH treatment. *N* = 8. *p < 0.5; **p < 0.01.

Proliferative responses of DLN cells from VEH-treated arthritic animals were elevated compared with DLN cells from non-arthritic rats (light gray horizontal bars; Figure 6A). Additionally, DLN cell proliferation in VEH-treated rats was reduced 1.5-fold compared with the spleen (Figure 4A), but 19- or 6.7-fold higher than for PBMCs (Figure 2A) or MLN cells (Figure 8A), respectively. *In vivo* treatment with TERB, PHEN, or PT dramatically (>600%) reduced DLN cell proliferation compared with the VEH-treated arthritic animals (Figure 6A; *p* < 0.001). In VEH-treated arthritic rats, IL-2 concentrations from DLN cell cultures were similar to spleen cell cultures (Figure 6B), and much lower than PBMC cell cultures (Figure 4B). Treatment with TERB or PHEN tended to increase IL-2 concentrations (*p* < 0.1; Figure 6B), but PT treatment significantly elevated IL-2 production compared with DLN cells from VEH-treated arthritic rats (*p* < 0.05) (Figure 6B).

### Adrenergic interventions differentially affected *ex vivo* cytokine secretion in DLN cells

**Figure 7:**
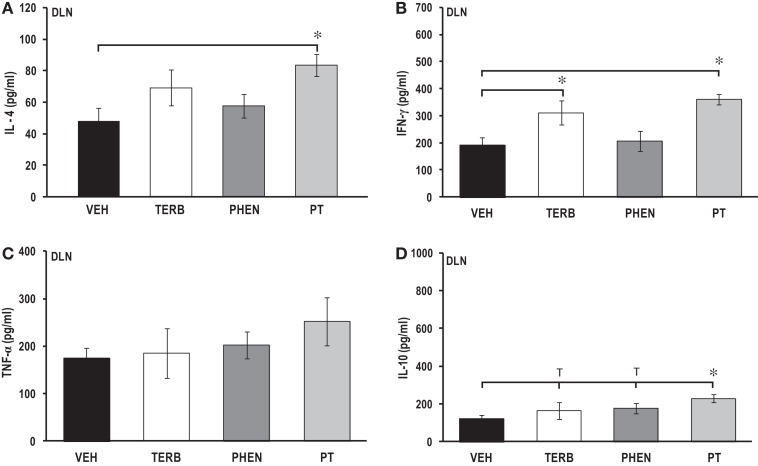
**Cytokine production by draining lymph node (DLN) cells after 24 h of culture**. Animals were treated with twice-daily i.p. injections of vehicle (VEH, black bars), terbutaline (TERB, white bars), phentolamine (PHEN, dark gray bars), or phentolamine and terbutaline (PT, light gray bars) initiated 12 days after adjuvant challenge. **(A)** IL-4 production was augmented by treatment with PT over that found in VEH controls. *N* = 8. *p < 0.05. **(B)** IFN-γ concentrations were higher in TERB- and PT- than with VEH-treated rats. *N* = 8. *p < 0.05; **p < 0.01. **(C)** TNF-α levels were similar in all groups. *N* = 8. *p* < 0.05. **(D)** IL-10 secretion was elevated in rats treated with PT over that seen with VEH treatment. *N* = 8. *p < 0.05.

*In vivo* administration of PT significantly increased IL-4 levels over levels in VEH-treated arthritic rats (Figure 7A; *p* < 0.05), but there was no effect of TERB or PHEN treatment on IL-4 concentrations. Interestingly, TERB and PT, but not PHEN, increased IFN-γ production in DLN cells compared with VEH treatment (*p*_TERB_ < 0.05; *p*_PT_ < 0.01) (Figure 7B). In contrast, none of the adrenergic treatments altered TNF-α production in DLN cells compared with VEH-treated arthritic rats (Figure 7C). PT treatment significantly increased the IL-10 concentration compared with VEH-treated arthritic rats (*p*_PT_ < 0.05) (Figure 7D), but treatment with TERB or PHEN had no effect compared with VEH-treated arthritic rats.

### Adrenergic therapies suppressed proliferation in MLN cells without affecting IL-2 production

**Figure 8:**
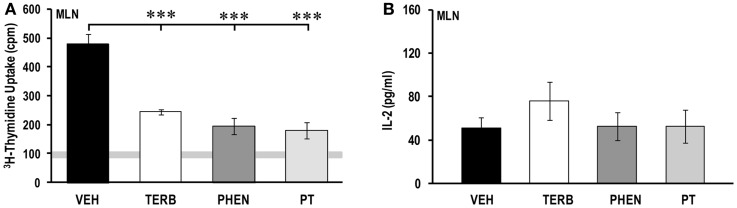
**Proliferation of mesenteric lymph node (MLN) cells and IL-2 production**. Animals were treated with twice-daily i.p. injections of vehicle (VEH, black bars), terbutaline (TERB, white bars), phentolamine (PHEN, dark gray bars), or phentolamine and terbutaline (PT, light gray bars) initiated 12 days after adjuvant challenge. **(A)** All drug treatments suppressed proliferation of MLN cells compared with VEH treatment. Horizontal light gray bar demonstrates non-specific background. **(B)** IL-2 production did not differ between treatment groups. *N* = 8. ***p < 0.001.

Thymidine incorporation by MLN cells was greater for all treatment groups than unstimulated non-arthritic rats (indicated by gray horizontal bars; Figure 8A). Drug-treated MLN cultures had lower cell proliferation (>600%) than VEH-treated rats (*p* < 0.001). Proliferative responses in MLN (Figure 8A) were 10-fold lower than observed for spleen (Figure 4A), but 2.8-fold greater than observed for the PBMCs (Figure 2A). IL-2 production was low, with no differences between treatment groups (Figure 8B).

### Adrenergic treatment-induced suppression of pro-inflammatory and Th cytokine production in cultured MLN cells

**Figure 9:**
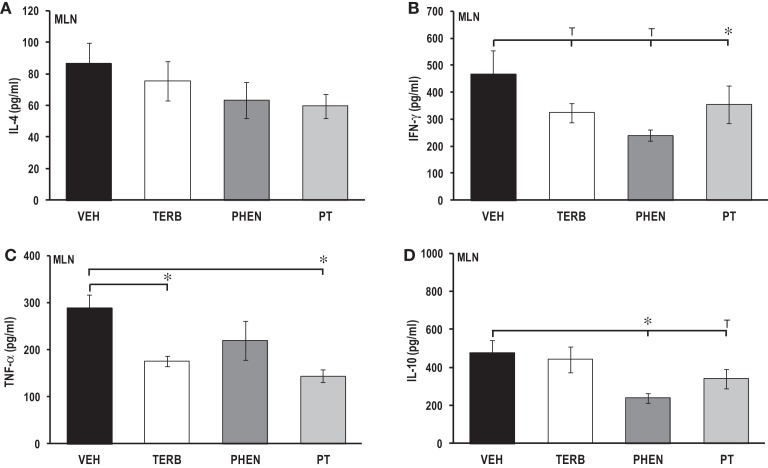
**Cytokine production by mesenteric lymph node (MLN) cells after 24 h of culture**. Animals were treated with twice-daily i.p. injections of vehicle (VEH, black bars), terbutaline (TERB, white bars), phentolamine (PHEN, dark gray bars), or phentolamine and terbutaline (PT, light gray bars) initiated 12 days after adjuvant challenge. **(A)** IL-4 production was comparable in all groups. *N* = 8. **(B)** IFN-γ concentrations were lower in PT- than with VEH-treated rats. *N* = 8. *p < 0.05. **(C)** TNF-α levels were reduced with TERB and PT treatment compared with VEH controls. *N* = 8. *p < 0.05. **(D)** IL-10 secretion was suppressed in rats treated with PHEN over that seen with VEH treatment. *N* = 8. *p < 0.05.

There were no significant effects of any of the drug treatments on IL-4 concentrations in MLN cell cultures compared with VEH treatment (Figure 9A). IL-4 concentrations from MLN cell cultures were similar to levels observed in spleen (Figure 5A), but almost double values observed in PBMC or DLN cell cultures (Figures 3A and 7A, respectively). *In vivo* treatment with TERB or PHEN alone had no effect on the IFN-γ production in MLN cells compared with the VEH-treated arthritic rats (Figure 9B). However, PT treatment significantly reduced IFN-γ production compared with VEH-treated arthritic rats (*p* < 0.05). TERB and PT treatment reduced TNF-α production compared with VEH treatment (Figure 9C; *p* < 0.05); however, PHEN treatment alone had no effect on TNF-α secretion. *In vivo* treatment with TERB did not significantly alter IL-10 production compared with VEH-treated controls; however, PHEN treatment inhibited IL-10 production by MLN cells compared with VEH-treated arthritic rats (Figure 9D; *p* < 0.05). There was a trend (*p* < 0.1) for lower IL-10 levels in the arthritic rats receiving PT treatment compared with the VEH treatment. IL-10 production in MLN cells (Figure 9D) from VEH-treated arthritic rats was 17-, 4-, and 4-fold greater compared with PBMC, spleen, and DLN levels, respectively (Figures 3D, 5D, and 7D, respectively).

### Lymphoid organ-dependent effects of adrenergic therapies on Th1/Th2 cytokine ratios

**Figure 10:**
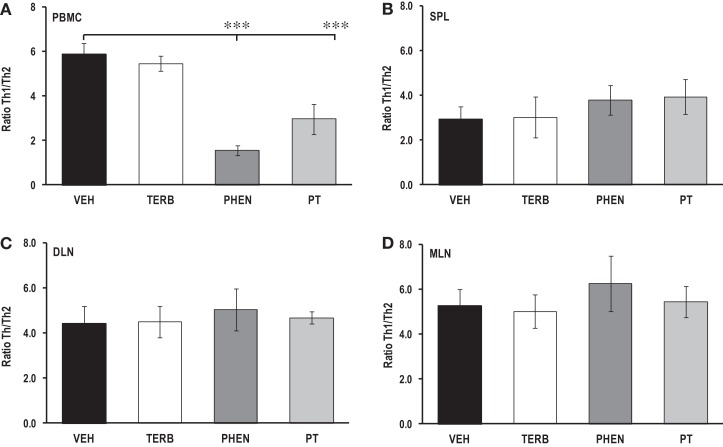
**Ratios of Th1 to Th2 cytokines in secondary immune compartments**. Animals were treated with twice-daily i.p. injections of vehicle (VEH, black bars), terbutaline (TERB, white bars), phentolamine (PHEN, dark gray bars), or phentolamine and terbutaline (PT, light gray bars) initiated 12 days after adjuvant challenge. **(A)** PBMC: PHEN and PT treatments lowered the Th1/Th2 cytokine ratio in PBMC cultures compared with VEH treatment. PT Th1/Th2 cytokine ratios were higher than with PHEN treatment. *N* = 8. ***p < 0.001. **(B)** Spleen:Th1/Th2 cytokine ratios did not differ between AA rats treated with any of the drug treatments compared with VEH treatment. **(C)** DLN, similar ratios were found in all groups. **(D)** MLN, no differences in Th1/Th2 cytokine ratios were found between treatment groups.

Th1/Th2 cytokine ratio (IFN-γ/IL-4) from the PMBCs did not differ between arthritic rats treated with TERB or VEH (Figure 10A); however, treatment with PHEN or PT reduced the Th1/Th2 cytokine ratio compared with VEH-treated rats (*p* < 0.001). In contrast, adrenergic drug treatments failed to affect the Th1/Th2 ratio in spleen, DLN or MLN cell cultures compared with arthritic rats treated with the VEH (Figures 10B–D, respectively).

### Lymphoid organ-dependent effects of adrenergic therapies on pro-inflammatory/anti-inflammatory cytokine ratios

**Figure 11:**
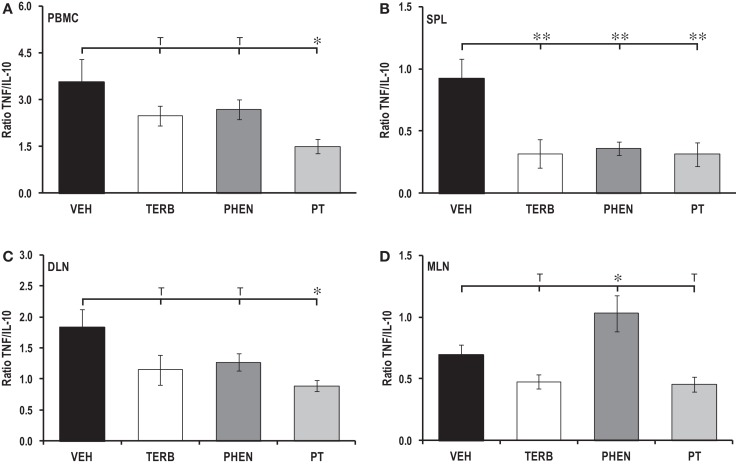
**TNF/IL-10 ratios in secondary immune compartments**. Animals were treated with twice-daily i.p. injections of vehicle (VEH, black bars), terbutaline (TERB, white bars), phentolamine (PHEN, dark gray bars), or phentolamine and terbutaline (PT, light gray bars) initiated 12 days after adjuvant challenge. **(A)** PBMC: PT treatments lowered the TNF/IL-10 ratio in PBMC cultures compared with VEH treatment. *N* = 8. *p < 0.05. **(B)** Spleen: all drug treatments lowered the TNF/IL-10 ratio compared with VEH treatment. *N* = 8. **p < 0.01. **(C)** DLN, PT suppressed the TNF/IL-10 ratio compared with VEH controls. *N* = 8. *p < 0.05. **(D)** MLN, the TNF/IL-10 ratio was greater in rats treated with PHEN than in VEH controls. *N* = 8. *p < 0.08.

The ratio of pro- to anti-inflammatory cytokine production (TNF-α/IL-10) in PBMCs and DLN cells revealed that while TERB or PHEN had minimal effects on the TNF-α/IL-10 ratio, PT treatment significantly reduced the ratio of TNF-α/IL-10 in both cell populations (*p* < 0.05, Figures 11A,C). In contrast, spleen cells from all three adrenergic therapies had reduced TNF-α/IL-10 ratios compared with VEH-treated arthritic rats (Figure 11B; *p* < 0.01). Interestingly, treatment with PHEN increased the ratio of TNF-α/IL-10 over VEH control levels in MLN cell cultures (Figure 11D; *p* < 0.05). Finally, there was a trend (*p* < 0.1) for reduced TNF-α/IL-10 ratios in the MLN cell cultures following *in vivo* TERB or PT treatment.

## Discussion

In this study, we confirm and extend our previous findings of the disease-modifying effects of a β_2_-AR agonist and/or α-AR antagonist treatment in AA after disease onset. Consistent with previous findings, TERB, PHEN, or PT dramatically ameliorates AA in male Lewis rats when treatment begins at disease onset (12). Our data extend these findings to show that all drug treatments suppressed proliferative responses in cell cultures from all lymphoid tissues examined. However, IL-2 production is not coupled with proliferation regardless of the adrenergic therapy. Immune cells in secondary lymphoid organs and peripheral blood are largely non-responsive to treatment with the β_2_-AR agonist. These findings support that the SNS loses its ability to regulate immune cell functions via normal β-AR signal transduction in secondary lymphoid organs in AA and by extension RA. Importantly, the exceptions to these findings are the dramatic β_2_-AR-mediated increase in spleen cell IL-10 and DLN cell IFN-γ production, respectively. Surprisingly, treatment with the α-AR antagonist produces greater changes in cytokine profiles than targeting β_2_-ARs, suggesting a shift in the class of AR that predominantly regulates immune function during chronic disease. In sites not expected to elicit robust immune responses to CFA challenge (i.e., PBMCs and MLN cells), PHEN and PT suppresses IFN-γ and TNF-α cytokine production. In spleen cells, the major effect of PHEN or PT was to drive IL-10 and suppress TNF-α production. This cytokine profile is expected to suppress inflammation and drive Th2 responses, which are consistent with improved disease outcome of PHEN or PT treatment. In contrast to single drug treatments, PT increases IL-4 and IL-10 production in DLN cells, which is expected to suppress inflammation and promote Th2 immunity. This difference in cytokine profiles may account for PT having the greatest ameliorating effects on disease, particularly its bone sparing effects.

All adrenergic therapies reduce disease severity. Radiographs and scoring provide support for a direct and/or indirect effect of adrenergic drugs on all scoring components used to assess soft tissue swelling (i.e., inflammation) and bone destruction/remodeling in the arthritic hind-limbs. Direct effects of adrenergic agents have been reported for both cartilage and bone. Both chondrocytes and bone cells (osteoclasts and osteoblasts) express functional ARs (26, 27) and can alter their growth and resorption activity (28–31). Additionally, immune cells (i.e., macrophages, T, B, and NK cells) and their products (i.e., TNF-α, IL-1, IFN-γ, and IL-2) coordinate changes in bone cell function and are regulated by ARs (32–34). Our findings support direct and/or indirect effects of TERB, PHEN, and PT treatments in ameliorating inflammation and joint destruction, and indicate these treatments are disease-modifying, anti-rheumatic drugs (DMARDs). X-ray scores suggest that TERB was more effective at reducing swelling than either PHEN or PT based on soft tissue assessment, in agreement with other reports demonstrating β_2_-AR-mediated anti-inflammatory effects (35–37). The β_2_-AR-mediated decrease in inflammation may be mediated by inhibiting macrophage TNF-α production and/or reducing reactive oxygen species (35–37). Surprisingly, PT was much less effective in reducing soft tissue swelling than specifically targeting β_2_-, or α-ARs. This finding suggests opposing actions of α- and β-ARs in the inflamed hind limbs. Whether this interaction involves multiple cell types and/or direct or indirect interplay between α- and β-ARs in the same cell types is unclear. Provocative new data (38, 39) indicate that β- and α-AR subtypes can form heterodimers that affect downstream signaling to regulate the inflammatory response. Similar research is warranted in our model to better understand β- and α-AR-mediated mechanisms regulating soft tissue swelling.

Immune cells cultured from lymphoid organs of CFA-challenged rats proliferate *ex vivo* without any additional immune challenge, an approach that better mimics the *in vivo* setting. Immune cell proliferation was most pronounced in DLNs and spleen. This finding is consistent with reports of persistent high antigen load in these tissues (40) and the significant involvement of both the DLNs and spleen in disease induction and immune dysregulation during the effector phase of the immune response. In response to CFA challenge, arthritogenic T cells that can transfer disease to naïve rats are generated in the spleen and DLNs (41). In agreement with this, we observed the greatest proliferative responses in DLNs and spleen cells, whereas proliferation was barely above background in PBMCs and MLN cells. TERB, PHEN, or PT treatment significantly reduced cellular proliferation in the spleen, DLN, and MLN, but had no effect on PBMC proliferation. This is consistent with the normalization of spleen weights after adrenergic treatments and with secondary lymphoid organs being sites of lymphocyte clonal expansion.

The reduced proliferative responses with TERB treatment are also consistent with other studies that demonstrate β_2_-AR agonists and norepinephrine treatment reduce proliferation of antigen- and mitogen-challenged lymphocytes from secondary immune organs [reviewed in Ref. (21)]. Upon antigen challenge, T cell proliferation is promoted by the binding of IL-2 to the IL-2R expressed on T cells [reviewed in Ref. (21)]. Numerous studies have shown that norepinephrine/epinephrine decrease the proliferative responses in T cells by a β_2_-AR-mediated reduction in IL-2 production following mitogen, anti-CD3 antibody, or antigen challenge (21). However, TERB treatment had no significant effects on IL-2 in our study, despite the β_2_-AR-mediated reduction in proliferation. These findings indicate that IL-2 does not drive the lymphocyte proliferation observed in secondary lymphoid compartments during the effector phase of AA, and that TERB-induced decreases in lymphocyte proliferation are not mediated by IL-2. Reports that chronic secretion of TNF-α can cause IL-2 deficiency at sites of inflammation (42–44) is consistent with our high TNF levels, low IL-2 production and uncoupling of IL-2-induced proliferation. Moreover, our findings are consistent with T cell hyporesponsiveness to T cell receptor (TCR) engagement (42), with a switch from an IL-2-to-inflammatory-driven immune response that suppresses IL-2 gene transcription (44). Finally, the inability of TERB to reduce IL-2 production in spleen and DLN cells from AA rats is consistent with recent findings from our lab that β_2_-ARs are uncoupled from cAMP (18). Others have reported that IL-2 decreases β-AR density, removing a negative control over cellular proliferation (45). Similarly, in certain cell populations, IL-1β can impair β_2_-AR coupling with adenylate cyclase (46, 47). Future studies are needed to determine the mechanisms for the β_2_-AR-mediated suppression of proliferative responses observed in secondary lymphoid organs in AA rats.

Surprisingly, treatment with the α-AR antagonist also reduced lymphocyte proliferation, an effect also observed when combined with TERB. These findings indicate that norepinephrine interaction with immune cell α- and β-ARs have opposing influences on lymphocyte proliferation, with α-ARs promoting and β-ARs opposing lymphocyte expansion during the effector phase of AA. Studies examining the effects of α-AR agonists on lymphocyte proliferation have reported increased, decreased, or no effect [reviewed in Ref. (48)]. Given the current evidence to date, lymphocytes express primarily β_2_-ARs, while innate immune cells appear to express β_2_-, α_1_-, and α_2_-ARs. Thus, effects of the α-AR antagonist, PHEN, on lymphocyte proliferation are likely mediated indirectly via α-AR-induced changes in functions of innate immune cells, such as macrophages, and/or by altering norepinephrine availability through presynaptic α_2_-ARs. Given the multiple potential targets of α-AR ligand, the mixed results regarding α-AR-mediated effects on proliferation is not surprising. Discrepancies regarding the direction of the impact of α-ARs-targeting drugs on lymphocyte proliferation are likely due to experimental differences, such as the type of antigen challenge, *in vivo* or *in vitro* drug administration, strain differences, and timing of administration relative to immune challenge. Collectively, our findings support that in contrast to β_2_-ARs, α-ARs suppress lymphocyte cell proliferation during the effector phase of AA, an effect that is blocked by α-AR antagonists during the effector phase. The extent to which the effects of TERB, PHEN or PT on proliferative responses contributes to disease reduction is unclear. However, inhibition of clonal expansion of arthritogenic T cells would be expected to reduce disease severity. Future studies will explore this possibility, as well as, the cell types targeted by these adrenergic treatments.

In contrast to TERB, PHEN treatment increases the production of IL-2 in spleen cells. Interestingly, PT also elevated IL-2 in DLN cells, but had no effect in other lymphoid compartments. The importance of these findings for reduced pathology is not clear. IL-2 is a pleiotropic cytokine. Besides its potent T-cell growth factor activity, IL-2 is essential for promoting the differentiation of Th1 (49) and Th2 cells (50). While IL-2 inhibits Th17 (51) and T follicular helper (Tfh) cell development (52), it does promote Th17 cell expansion once these cells develop (53). IL-2 is also involved in activation-induced cell death (AICD), which is important for homeostasis and the elimination of potentially harmful auto-reactive cells, at least in part by a Fas and FasL-dependent mechanism (54). IL-2 promotes antibody production and proliferation by B cells (55) and drives the development of CD4+FOXP3+ regulatory T cells (Treg cells), which have suppressor functions and mediate tolerance (56–58). Thus, IL-2 has broad essential biological actions, not only driving T cell proliferation and modulating effector cell differentiation, but also limiting potentially dangerous autoimmune reactions. Further research is required to understand the impact of α- and β-AR-mediated changes in IL-2 production on disease outcome in our disease model.

Regarding IL-2 and leukocyte proliferation responses, our findings are consistent with reports of increased SNS activity, decreased IL-2 production, and reduced proliferative responses in RA patients. Serum levels of IL-2 are reduced in patients with active disease, particularly in those with extra-articular manifestations (59, 60). Further, in RA patients mitogen-stimulated PBMCs produce less IL-2 and proliferate less robustly than PBMCs from controls (59). Low IL-2 and proliferative responses inversely correlate with disease activity and positively correlate with the proportion of CD4+ T cells (59), suggesting their importance in the pathophysiology of RA. Moreover, monocytes may play a role in regulating IL-2, as their depletion and partial reconstitution increases IL-2 production and proliferation in RA patients. Circulating cytokines that are indicators of general immune activation (including IL-2) increase prior to disease onset in RA patients (61), consistent with their importance in disease onset. Our data showing the ability of an α-AR antagonist or combined β_2_-AR agonist and α-AR antagonist treatment to increase splenic and DLN IL-2, respectively, indicate the complexity of regulatory mechanisms for this cytokine by the SNS. IL-2 is at the “cross-roads” of both effector T-cell responses and tolerance [reviewed in Ref. (62)], and the development of lethal autoimmune disease in IL-2 knock-out mice (63). We have speculated that this particular cytokine may be the key to explaining opposite effects of adrenergic agonists on disease severity when administered prior to vs. after disease onset (12). If this hypothesis is true, then understanding of the mechanisms for these contradictory effects of adrenergic treatments relative to disease onset has implications for prevention and therapeutic treatment of RA.

In the present study, TERB treatment had no effect on IFN-γ production in spleen, MLN and PBMC, despite the well accepted role of norepinephrine via β_2_-ARs to inhibit and enhance the production of the Th1 and Th2 cytokines, IFN-γ and IL-4, respectively (64, 65). The inability of TERB treatment to impact Th cell IFN-γ and IL-4 production in spleen, MLN, and PBMCs are consistent with findings by Heijink and coworkers (66) demonstrating that polarized Th1 and Th2 cells are less responsive to negative feedback by receptors coupled to the adenylate cyclase/cAMP pathway compared with freshly isolated T cells. Sanders and coworkers (67) also showed that in freshly isolated CD4+ T cells, a β_2_-AR agonist could activate the cAMP pathway to increase IL-2 and IFN-γ, however, this response is attenuated in differentiated Th1 and Th2 cells. In these studies, specialized Th subset cells were stimulated by anti-CD3/anti-CD28 or *in vitro* differentiation of Th0 cells under Th1 or Th2 polarizing conditions. Similar to the differentiated Th cells, we also observed that treatment with β_2_-AR agonists is no longer able to induce an increase in intracellular cAMP in splenocytes from arthritic rats 28 days post-adjuvant challenge (18). Further, lymphocyte β_2_-ARs in spleen cells from arthritic rats were phosphorylated at a site known to induce receptor desensitization. Our findings are consistent with the loss of control over cytokine production by the β_2_-AR-coupled cAMP pathway in Th effector cells after CFA challenge. These findings are also consistent with the differential effects on disease outcome in AA rats when a β-agonist is administered at different times across the time course of AA. Thus, differential effects of the agonist after CFA challenge and after disease onset may be due to targeting of differentiating Th0 cells versus fully differentiated Th cells.

Despite the known inhibitory effects of β_2_-AR agonists on Th1 cell production of IFN-γ (64, 65), TERB treatment in arthritic rats elevated IFN-γ production in the DLNs. This finding suggests that β_2_-AR signaling is not only uncoupled from its normal cAMP-PKA pathway, but also switches receptor coupling to an alternate second messenger. Recent findings from our lab indicate that in DLNs, β_2_-ARs are phosphorylated at a site known to switch signaling from cAMP to mitogen-activated protein kinase (MAPK) pathways (18). In AA rats, DLNs drain a site of chronic inflammation, and thus, are exposed to high concentrations of inflammatory cytokines. This may explain, in part, the different β_2_-AR responses in spleen and DLN cells. Consistent with this hypothesis, IL-1β is reported to cause a concentration- and time-dependent decrease in responses of airway smooth muscle cell and cardiac myocyte to a β-AR agonist that is mediated by uncoupling β-AR from Gs-induced activation of adenylyl cyclase (47, 68, 69). This response was accompanied by an increase in membrane Gi expression (69), a G-protein coupled with MAPK activation. The DLNs also receive a much higher antigen load than other secondary lymphoid organs after base of the tail CFA challenge. Additionally, the slow release of the *M. butyricum* cell wall components from the adjuvant may result in the persistent sub-optimal challenge in the DLNs. Interestingly, low persistent challenge with environmental adjuvant exposure has been linked to failed tolerance and the development of autoimmunity (70).

Neither TERB nor PHEN treatment altered IL-4 production in any of the tissues compared with VEH-treated arthritic rats. Our findings are consistent with studies showing that treatment with either norepinephrine or TERB induces IL-4 production from Th2 clones or Th2 effector cells derived from β_2_-AR-stimulated naïve CD4+ T cells (67, 71, 72). In contrast, other studies have shown that elevations in cAMP were able to increase IL-4 production in Th2 cells (66). It is unlikely that β_2_-ARs directly mediate increased IL-4 production by Th2 cells as the differentiation of Th0 to Th2 cells induces the loss of β_2_-AR expression (67, 71, 72). Notably, PT treatment increased IL-4 production in the DLNs. Whether this is a direct or indirect result of TERB or PHEN, the result of an interaction between the two drugs on Th2 cell IL-4 production, or due to TERB- or PT-induced effects on other immune cell populations will require additional studies.

The most dramatic effects of PHEN treatment during the effector phase were the reductions of IFN-γ and IL-2 in the PBMCs. Since T cells do not normally express α-ARs, the effects of PHEN or PT on IL-2 or IFN-γ cytokine production could be indirect. PHEN binding to α_2_-ARs present in presynaptic terminals is expected to increase availability of norepinephrine for interaction with T cell β_2_-ARs. A change in circulating norepinephrine due to spillover from organs that are innervated and altered epinephrine release from the adrenal medulla could alter the circulating concentrations of norepinephrine and epinephrine. If this occurs, then circulating T cell β_2_-AR stimulation could induce changes in T cell cytokines in this compartment. However, since TERB had no effect on PBMC IFN-γ release, the decreased IFN-γ production observed following PHEN or PT treatment is unlikely to be mediated indirectly through presynaptic α_2_-ARs. Alternatively, these treatments could be targeting α-ARs expressed in monocytes to alter their production of cytokines that then induce changes in T cell cytokine production. This notion is consistent with a report by Heijnen and coworkers (73) showing functional α_1_-ARs in peripheral blood leukocytes (presumably monocytes) of patients with polyarticular juvenile RA. Finally, the altered PBMC T cell cytokine production observed after PHEN or PT treatment could alter the T cell subtypes that enters the circulation, increasing subtypes that produce higher amounts of IFN-γ and IL-2. Mechanisms responsible for the effects of PHEN treatment on IFN-γ and IL-2 production in PBMCs during the effector phase of AA requires further investigation.

In this study, TERB or PHEN treatment did not alter production of the pro-inflammatory cytokine, TNF-α in spleen, lymph nodes, or PBMCs in arthritic rats after disease onset. This is in contrast to reports that β_2_-AR agonists and α-AR antagonists inhibit macrophage TNF-α production, respectively [reviewed in Ref. (74–77)]. These findings suggest an impairment of signaling via both β_2_- and α-ARs that occurs during the effector phase of AA. Interestingly, treatment with PT significantly reduced production of TNF-α in the spleen, MLN, and PBMCs. It is not clear why the combination drug treatment reduces TNF-α, in the absence of effects by the individual treatments, but the findings suggest interactions between β_2_- and α-AR signaling. Collectively, these findings indicated that SNS signaling to immune cells via β_2_- and α-ARs is impaired, or induces alternative second messengers in monocytes/macrophages in these organs in rats with established AA.

The greatest effect of TERB and/or PHEN on cytokine production was observed for splenic IL-10 production, where all three adrenergic treatments induced a significant increase in IL-10 production. This finding is consistent with the known effects of β_2_-AR agonists and α-AR antagonists to increase IL-10 production (78, 79). The combination treatment increased IL-10 production to the same extent as treatment with each drug alone, suggesting that the effects of each of the components were neither additive nor synergistic. Thus, at least in the spleen, these receptors are capable of inducing production of second messengers, despite the lack of AR-induced responses for TNF-α production. In DLNs, IL-10 production increased significantly only after treatment with combined TERB and PHEN, although there was a trend for increased IL-10 with each individual adrenergic drug. We observed no effect for any of the adrenergic treatments in IL-10 production by PBMCs. In contrast, PHEN significantly reduced IL-10 levels in the MLNs, an effect that is inconsistent with reports that α-AR agonists suppress IL-10 production (78). Thus, the pattern of IL-10 production after adrenergic treatments was dependent upon the secondary lymphoid tissue examined.

Data supports that sympathetic dysfunctions significantly alter the balance between Th1-Th2 cell differentiation in RA (18, 80). RA is dominated by a Th1 response with selective accumulation of Th1 cells within the synovial compartment (81). RA and juvenile chronic arthritis co-exist with chronically elevated sympathetic activity [reviewed in Ref. (8)]. We and others have demonstrated that sympathetic innervation of the lymphoid organs in arthritic rodents (14, 15) and in arthritic joints of RA patients (17, 82) are lost and/or reorganized. Indeed, in this study, treatment with a β_2_-AR agonist fails to shift T lymphocyte cytokine responses toward a Th2 profile in any of the secondary lymphoid organs of AA rats. However, *in vivo* treatment with the α-AR antagonist shifts the *ex vivo* cytokine profile from Th1 to a Th2 response, indicating that α-AR stimulation inhibits this shift. These findings are consistent with a recent report showing that catecholamines fail to shift T cell cytokines from a Th1 to a Th2 profile in PBMCs from RA patients, as seen in healthy donors (83). Thus, impaired sympathetic functioning is intimately linked with disease pathology.

Stimulating β_2_-ARs and/or blocking α-ARs reduces the ratio of pro-to-anti-inflammatory cytokine production by PBMCs, spleen, and DLN cells. The reduced pro-to-anti-inflammatory cytokine ratios are consistent with the well-known effects of β_2_-AR stimulation or α-AR blockade to inhibit TNF-α and promote IL-10 production in macrophages and dendritic cells (74–79). Spleen cells displayed the greatest drug-induced changes in anti-inflammatory profiles, largely due to greater IL-10 production. In contrast, TERB or PHEN treatment, tend to induce an anti-inflammatory secretory profile in PBMCs and DLN cells. However, dual drug treatment that augment β_2_- and dampen α-AR activation-induced anti-inflammatory profiles. These changes were due to reduced TNF-α secretion in PBMCs and increased IL-10 in DLN cells. Therefore, TERB, PHEN, or PT-induced anti-inflammatory profiles may be responsible, at least in part, for improved disease outcomes in our study.

Interestingly, PHEN treatment induced a pro-inflammatory cytokine profile in MLN cells, an effect due to lower IL-10 in the absence of an effect on TNF-α production. Beta_2_-AR stimulation inhibited TNF-α secretion, but was unable to significantly reduce the pro-to-anti-inflammatory ratio. Thus, blocking α-AR activation in the MLNs had an opposing effect on IL-10 production to that seen in the spleen. The reason for this difference may lie in the cellular source of IL-10 in these immune organs, but requires further investigation. Moreover, our data suggest that activation of either α- or β-ARs have anti-inflammatory effects in the MLNs. Why our drug treatments differentially affected inflammatory drive depending on the specific lymphoid tissue is unclear and warrants further study. Perhaps these differences reflect the unique functions of each lymphoid organ/tissue in response to the CFA challenge and the site of injection. The primary function of DLNs is to respond to the intradermal challenge, whereas the spleen participates in the systemic response. As part of the systemic response in AA, the spleen filters the blood, and is a major source of arthritogenic T cells that infiltrate affected joints. In contrast, the blood serves as a conduit for trafficking cells of the immune system. Finally, the MLNs are most responsive to antigens that travel in lymphatic vessels from the gut. Collectively, our findings indicate that the SNS maintains the ability to inhibit (via β_2_-ARs) or promote (via α-ARs) inflammation in PBMCs, splenocytes, and DLN cells, but not in MLN cells.

Body weights are reduced 28 days after disease induction. This finding concurs with the well-documented AA-, CIA- and RA-induced cachexia (loss of body mass without altered diet or increased activity) due to inflammation that causes hypermetabolic protein and fat breakdown, skeletal muscle wasting, and to a lesser extent, lost white adipose tissue mass (12, 84–86). Moreover, treatment with PHEN, TERB, or PT significantly reduced disease-associated cachexia. The significance of these findings is underscored by cachexia occurring in ~2/3rds of all RA patients, and is a major contributor in greater morbidity and mortality in RA patients (84, 87, 88). Here, we did not monitor food intake, but other studies have shown that this factor is not contributory to disease-induced weight loss (89). However, inflammation indirectly reduces cellular mass by protein breakdown due to activation of the ubiquitin–proteasome pathway (90) and lipolysis (86). These effects are mediated at least in part by TNF-α (91). Adrenergic therapies in our study suppresses *ex vivo* TNF-α production and the pro- to anti-inflammatory ratios in a treatment- and immune organ-specific manner, consistent with a role for inflammation in rheumatoid cachexia.

In summary, it is clear that the critical functions of the SNS to regulate lymphocyte proliferation, Th1/Th2 cell differentiation, and inflammation are impaired/altered in AA rats, due to altered β_2_- and α-AR functions. Selective α-AR blockade and/or β-AR activation in AA suppresses *ex vivo* proliferation, but is independent of sympathetic regulation of IL-2 production in AA. This is true in all immune organs examined, except PBMCs, and is consistent with the function of blood as a conduit for immune cell trafficking. Adrenergic treatments affect Th1/Th2 cytokines differently in every tissue examined in AA, perhaps reflecting the different immune functions of each organ. Most striking was the β_2_-AR-mediated increase in IFN-γ in the DLNs and lack of a β_2_-AR-mediated effect on IFN-γ in the spleen, particularly since arthritogenic T cells are generated at both sites (92, 93). Regardless of these differences, the overall outcome is a failure to drive Th2-type cytokine expression via β_2_-AR stimulation, and thus, loss of a negative feedback mechanism for regulating cellular immunity. These findings are consistent with reports of disease-induced elevation in sympathetic tone (8), and high norepinephrine concentration-dependent β_2_-AR down-regulation, desensitization, and/or shifts in G-protein coupling (18, 82, 94, 95). Indeed, we have previously discovered differences in phosphorylation of β_2_-ARs in DLN and spleen cells collected from AA rats that may explain the IFN-γ findings in this study (18). Further, essential SNS regulation of innate immunity, via β_2_- and α-ARs, is also impaired as demonstrated, with inability of β_2_-AR stimulation or α-AR blockade to reduce TNF-α and increase IL-10 production in DLN cells. Stimulation of β_2_-ARs similarly fails to reduce TNF-α production in the spleen cells.

Collectively, our findings indicate disrupted β_2_-ARs, but not α-ARs signaling in AA, which consequently derails sympathetic regulation of lymphocyte expansion, Th cell differentiation, and inflammation required for immune system homeostasis. The clinical relevance of our findings is several-fold. First, they underscore the complexity of the disease’s relevant immune functions, their regulation by the SNS and the limitations of peripheral blood to decipher complex pathology. Secondly, distinct differences in SNS-immune interactions in spleen and lymph nodes likely reflect functional and microenvironmental differences of these immune organs, which currently are under appreciated and sources of discrepancies, inconsistencies, and confusion in the literature. Thus, relevant animal models for RA and novel approaches are required to elucidate both immune and neural-immune mechanisms in disease pathology. Finally, disease-modifying and suppressive inflammatory effects of targeting β_2_- and α-ARs strongly support that combined AR-targeted therapies have great therapeutic potential to reduce joint destruction and inflammation and complications of RA, like cachexia. While promising, a greater understanding of the mechanisms by which these beneficial effects are realized is essential to evaluate therapeutic potential and guide clinical applications.

## Author Contributions

All authors made a substantial contribution to conceiving and/or designing of the research project, the acquiring, analyses, or interpretation of the data presented in this paper. All authors have contributed to writing of this paper and its intellectual content and critical evaluation of content. All authors have approved the final version of this paper, and are accountable for the accuracy and integrity of the research presented in this paper.

## Conflict of Interest Statement

The authors declare that the research was conducted in the absence of any commercial or financial relationships that could be construed as a potential conflict of interest.
